# Celestial Navigation: A State-of-the-Art Review Towards a Light Autonomous Underwater Vehicle (LAUV) Conceptual Application

**DOI:** 10.3390/s26154790

**Published:** 2026-07-28

**Authors:** Suzana Lampreia, Hugo Policarpo, Pedro P. de Almeida, Nuno R. Roboredo, Jorge M. Ruivo, Rafael B. Henriques, Victor Lobo

**Affiliations:** 1 Centro de Investigação Naval (CINAV), Portuguese Naval Academy, Base Naval de Lisboa, 2810-001 Almada, Portugal; suzana.paula.lampreia@marinha.pt (S.L.); pereira.almeida@marinha.pt (P.P.d.A.); rocha.roboredo@marinha.pt (N.R.R.); marcelino.ruivo@marinha.pt (J.M.R.); bagagem.henriques@marinha.pt (R.B.H.); sousa.lobo@marinha.pt (V.L.); 2 IDMEC, Instituto Superior Técnico, Universidade de Lisboa, 1049-001 Lisboa, Portugal; 3 Instituto de Plasmas e Fusão Nuclear (IPFN), Instituto Superior Técnico, Universidade de Lisboa, 1049-001 Lisboa, Portugal; 4 NOVA Information Management School (NOVA IMS), Universidade NOVA de Lisboa, Campus de Campolide, 1070-312 Lisboa, Portugal

**Keywords:** celestial navigation, astronomic navigation, astronavigation, autonomous underwater vehicle, GNSS-denied navigation, solar tracking, sensor fusion, LAUV

## Abstract

**Highlights:**

**Which are the main findings?**

- A state-of-the-art review of celestial navigation technologies identifies solar-based positioning as a technically feasible and strategically relevant complement to conventional Autonomous Underwater Vehicles, providing periodic absolute position fixes to correct Inertial Navigation System/Doppler Velocity Log drift and offering a credible independent fallback navigation in Global Navigation Satellite System-denied environments.
- A preliminary spatial layout for incorporating Sunto’s Solar Tracking Sensor and a Tracking Control Intelligence unit into a Light Autonomous Underwater Vehicle platform is proposed. This conceptual analysis illustrates the theoretical geometric feasibility of the module, establishing a baseline for future hydrodynamic validation.

**What are the implications of the main findings?**

- The proposed conceptual framework illustrates how celestial navigation can theoretically provide periodic absolute position fixes to correct Inertial Navigation System/Doppler Velocity Log drift, thus offering a credible Global Navigation Satellite System-independent fallback for long-endurance Autonomous Underwater Vehicle missions.
- The proposed ten-step development methodology establishes a reproducible Autonomous Underwater Vehicle platform integration framework applicable to future celestial navigation systems.

**Abstract:**

Global Navigation Satellite Systems (GNSS) are incompatible with the underwater domain as radiofrequency signals cannot penetrate the water column, leaving Autonomous Underwater Vehicles (AUVs) reliant on dead-reckoning systems that accumulate positional errors over time. When AUVs surface to reset their navigation, they face another challenge: GNSS itself is increasingly vulnerable to jamming and spoofing in contested environments. Automated Celestial Navigation (CN) has emerged as a promising alternative to other navigation methods, making it possible to derive the absolute position from observations of celestial bodies, entirely independent of human-made signals. This work provides a state-of-the-art review of automated CN technologies, focusing on the literature from 2020 onwards. The review covers Solar Tracking Sensors (STSs), star trackers and horizon detection algorithms and assesses their suitability for AUV integration through a structured SWOT analysis. Following this, a conceptual CN system based on Sunto’s STS is developed for the Light Autonomous Underwater Vehicle platform employing a proposed ten-step integration methodology. Computer-Aided Design models illustrate the conceptual setup, though hydrodynamic/structural verification remains subject to future work. Results suggest that solar-based CN can serve as a periodic absolute position corrector within a hybrid AUV navigation architecture, without requiring satellite infrastructure, which contributes towards a more resilient AUV navigation.

## 1. Introduction

Nowadays, maritime navigation systems depend heavily on Global Navigation Satellite Systems (GNSSs), a dependency with vulnerabilities that were largely invisible until jamming and spoofing incidents began to expose it [1]. Overreliance on GNSSs may constitute a significant potential point of failure, driving the need for other backup systems.

The problem is considerably worse underwater, where GNSS signals or any other radiofrequency signals cannot effectively penetrate the water column. This compels Autonomous Underwater Vehicles (AUVs) or Unmanned Underwater Vehicles (UUVs) to rely on internal dead-reckoning systems, Inertial Navigation Systems (INSs) and Doppler Velocity Logs (DVLs), or other terrain-following systems when the AUV is close to the ocean floor. While these systems have been demonstrated to be effective over short durations, they are inherently subject to cumulative error drift over time [2]. Therefore, long-endurance AUVs must periodically surface to establish a GNSS signal and reset their navigational drift. In the event of GNSS compromise or denial upon surfacing, the AUV risks losing its position and/or fail its mission.

Celestial Navigation (CN) (Also referred to as astronomic navigation or astronavigation. These terms are proper and widely accepted names for the practice of finding the position using the stars, sun, moon and planets. The names originate from slightly different linguistic roots but refer to the exact same ancient and enduring discipline), one of the oldest forms of wayfinding [3], is undergoing a contemporary resurgence as a potential solution to these modern challenges. With it, it is possible to achieve absolute global positioning by observing the position of celestial bodies such as the sun, moon, planets and stars relative to the horizon (or local vertical reference) and time. Recent advances in low-cost optical sensors, image processing algorithms and miniaturized star trackers have rendered the automation and miniaturization of CN equipment a viable outlook to address GNSS-denied positioning in AUVs [4,5].

Although automated CN systems have been used for a long time in satellites, spaceships and a few other specialized systems, they tended to be relatively expensive and quite bulky. In the context of a Light Autonomous Underwater Vehicle (LAUV) [6], the integration of a miniaturized optical sensor promotes a jam-resistant positional fix during its periodic surfacing maneuvers, complementing its reliance on GNSS, but the cost, size and integration within the LAUV poses challenging issues.

This paper provides a state-of-the-art review of current CN systems, aiming to identify the feasibility, existing limitations and future development paths required to successfully integrate CN into the next-generation LAUV. This objective is pursued by evaluating the literature and technological advances from 2020 onwards in automated star-tracking, horizon detection algorithms and hardware miniaturization.

Following this introduction, the work is organized as follows. Section 2 presents a state-of-the-art review of AUV navigation systems and recent advances in CN technologies, including a Strengths, Weaknesses, Opportunities and Threats (SWOTs) analysis that contextualizes CN within the broader navigation landscape. Section 3 describes the methodology developed for the conceptual integration of a CN system into the LAUV platform. Section 4 presents the main conceptual results, comprising sensor selection, hardware architecture and Computer-Aided Design (CAD) models. Section 5 discusses the findings in the context of the previous sections and Section 6 provides the final remarks and directions for future work.

## 2. Recent Advances in AUV Navigation: A Brief Review

AUV navigation relies both on hardware and software in which acoustic sensors, sonars, cameras, INS and/or different sensor combinations as well as algorithms and methods are crucial.

### 2.1. Common Navigation Systems (Hardware and Software)

AUV navigation relies on a diverse range of hardware and software solutions to provide reliable positioning in complex underwater environments where GNSS is unavailable. To establish a baseline for the recent advances discussed in this section, Table 1 provides an overview of the standard navigation technologies and auxiliary sensor inputs commonly employed in underwater platforms [7,8], highlighting their core capabilities and operational challenges.

A brief overview of these standard systems examines each technology category with emphasis on the specific limitations that CN is positioned to address, e.g., drift accumulation in INS/DVL systems, dependence on pre-deployed acoustic infrastructure and the inability to obtain absolute position fixes when GNSS is unavailable or compromised.

In practice, AUVs rely on INS fused with DVL for continuous positioning [2,5,6,7,8]. The combination works well over short missions, but position errors accumulate over time. It is this drift that CN may be designed to correct by providing a periodic absolute fix at surfacing [8]. Acoustic positioning systems such as Long BaseLine (LBL) and Ultra-Short Baseline (USBL) can supply absolute fixes, but their reliance on pre-deployed transponder arrays rules them out wherever infrastructure cannot be placed in advance [7,8]. Pressure sensors give reliable depth measurements, while sonar and optical sensors support obstacle avoidance and landmark-based localization, though both degrade with turbidity, light attenuation and variable sound speed [7,8]. Critically, none of these approaches can provide an absolute, satellite-free horizontal position fix, one of the gaps that CN addresses.

Building upon these established systems, recent research has increasingly focused on hybrid architecture and Artificial Intelligence (AI) to mitigate these persistent weaknesses.

Table 2 captures the AUV navigation work from 2020 to 2026 most relevant to the trends described above.

Table 2 makes clear that no single AUV navigation technology is universally adequate [9]. Research has consequently moved towards hybrid architectures that combine inertial sensors with complementary environmental inputs: visual odometry [11], underwater polarization [12] and geophysical referencing [13] have all been explored as ways to constrain dead-reckoning drift without satellite contact. Alongside this sensor diversification, AI-based filtering has become the dominant tool for managing the resulting data fusion complexity. Deep neural network approaches, specifically GRU-PF [10] and RBF [14], have shown that learning vehicle dynamics from historical data can outperform classical mathematical models, particularly under the nonlinear conditions that underwater vehicles usually encounter.

While these state-of-the-art works are designed for an inherently GNSS-denied underwater environment, one of their primary focuses remains on overcoming the physical limitations of the water column rather than addressing deliberate GNSS denial. Hence, there is an apparent research gap in the current underwater navigation literature regarding how AUVs handle tactical GNSS-compromised or actively spoofed environments during multi-domain operations. This gap highlights a substantial vulnerability when vehicles surface, a limitation that this paper addresses by introducing a resilient, celestial-based backup framework.

Among the GNSS-independent navigation alternatives reviewed, geomagnetic navigation [13] and underwater polarization navigation [12] stand out because they operate fully submerged, with no surfacing required. However, geomagnetic methods depend on pre-existing geophysical map databases and are limited by local magnetic anomalies, while polarization techniques are sensitive to water turbidity and require calibration for the underwater light field. CN avoids both issues as celestial bodies are universally visible from the surface, no database is needed, and no signal travels through the water column. This makes it a natural fit for the periodic surfacing events that are already built into LAUV mission profiles.

Nevertheless, despite the recent advancements in AI-enhanced filtering and hybrid sensor fusion, the core challenges of time-varying drift and environmental constraints in standard inertial and acoustic systems persist [8]. This underscores the necessity of adapting navigation architectures to specific mission profiles, particularly when GNSS availability is compromised or denied. Among the emerging alternatives, recent research increasingly points to CN as a robust candidate for providing the periodic absolute position fixes required to reset accumulated navigational errors [5,8,13,15].

### 2.2. Celestial Navigation

What was once a purely manual skill is now being rebuilt as an automated system being driven, in large part, by the recognition that GNSSs cannot be taken for granted [15,16]. Historically, the primary limitation of CN has been its reliance on human-centric manual observation, which is weather-dependent and highly prone to error. These issues persist even when operators use commercial smart devices [17].

To minimize these issues, recent research has placed emphasis on the implementation of full automation. The replacement of manual observations with automated optical cameras, star trackers and AI algorithms enables the autonomous identification of celestial bodies and the calculation of altitudes with high precision [4,18]. This hardware automation is heavily supported by adaptively robust filtering algorithms designed specifically to mitigate maritime observation noise [19].

Automation opens CN up to platforms where human observers were never a practical option. Recent solutions contemplate affordable vision-based strapdown algorithms [5], specialized solar-based celestial techniques and dedicated hardware such as continuous Solar Tracking Sensors (STSs) [20]. Similarly, the conceptual design and optimal Field of View (FOV) for these automated celestial cameras are undergoing active development for maritime navigation [21].

Applying CN to an AUV, however, introduces a layer of engineering challenges that has no counterpart in terrestrial or aerospace applications. As previously mentioned, one fundamental constraint is optical access. An AUV operating at depth has no line of sight to the sky, requiring it to surface or deploy a periscope/mast arrangement to perform celestial observations. This surfacing requirement imposes mission planning constraints and limits applicability in covert or deep-water operations. Once at the surface, sea state adds its own complications as horizon instability and vehicle motion introduce noise in altitude measurements in ways that simply do not arise for land-based or airborne users. The hardware must also survive the marine environment. Saltwater, biofouling, pressure cycling and vibration impose qualification demands. Finally, the intermittent nature of surfacing-based observations means CN cannot provide a continuous navigation stream, meaning it works as a periodic absolute position corrector within a hybrid architecture, resetting the dead-reckoning clock at each surfacing event. Put together, these constraints suggest that a fully automated CN system is more of an engineering problem than a theoretical one.

A summary of the highlights of some recent (2020–2026) relevant works on these topics is presented in Table 3.

Two other CN modalities are worth examining alongside solar tracking. Miniaturized star trackers, originally developed for aerospace altitude determination, achieve angular accuracies of 0.002–0.01 degrees through star pattern matching, making them highly competitive in positioning precision, though limited to night-time or low-light operation [22]. Polarization-based navigation, demonstrated in [12], takes a different approach. It uses polarized light field patterns to determine heading angles that, when fused with inertial information through strapdown navigation, enables AUV navigation with high accuracy in real underwater environments. One of its most operationally significant features is that it can work fully submerged, i.e., no surfacing is needed. This sets it apart from both solar and star-based systems. Table 4 provides a side-by-side comparison of these three systems.

### 2.3. SWOT Analysis of Celestial Navigation

This SWOT analysis, see Table 5, synthesizes evidence from the reviewed literature on CN as a resilient alternative and complementary navigation paradigm for AUVs, particularly in GNSS-denied or GNSS-compromised environments.

The SWOT analysis points to several practical engineering solutions that are worth mentioning. The most operationally limiting weakness is weather dependence. Pairing solar tracking with a star tracker extends usable coverage to night-time and low-cloud conditions in which mission planning tools can use historical weather data to schedule surfacing events at statistically favorable moments. The surfacing constraint itself can be partially addressed through periscope or mast-mounted sensor configurations, though both carry drag and covertness trade-offs that need to be weighed against mission requirements. Biofouling deserves to be treated as a design requirement from the outset rather than a maintenance afterthought. For it, anti-fouling coatings and self-cleaning dome geometries are well-established engineering solutions that may be specified at the sensor integration stage. Finally, the low update rate inherent to any surfacing-based system is best managed at the filter level. Both the GRU-PF [10] and RBF [14] frameworks reviewed in Section 2.1 were designed to handle heterogeneous, intermittent sensor inputs, making them natural candidates for integrating CN fixes as periodic absolute position resets within the existing INS/DVL pipeline, reducing cumulative drift between surfacing events.

Pursuing hybrid integration with INS, DVL and AI-driven filtering, based on what the reviewed literature consistently shows, is the most promising direction for making CN work reliably on vehicles like the LAUV.

## 3. Methodology for the LAUV’s Conceptual CN System

**Figure 1:**
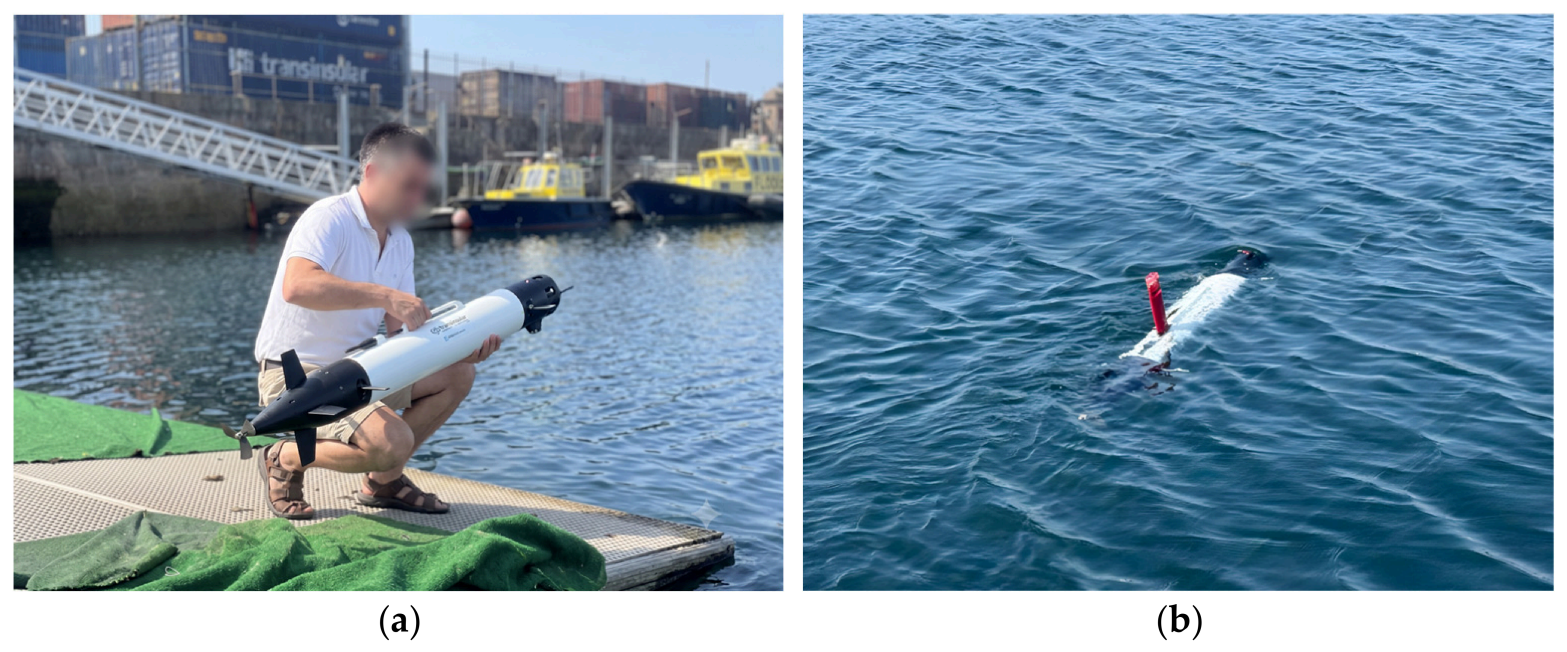
Photographs of an LAUV: (**a**) LAUV being man deployed; (**b**) LAUV in the water.

The LAUV is a lightweight, one-man-portable, modular platform system (see Figure 1) initially developed by the Underwater Systems and Technology Laboratory (LSTS) from the Porto University and subsequently co-developed in partnership with OceanScan-MST [6].

Its portability, reduced acquisition cost and broad payload compatibility position it as an effective, economical and low-risk experimental platform for AUV research programs and the prototyping of novel concepts of operation.

**Figure 2:**
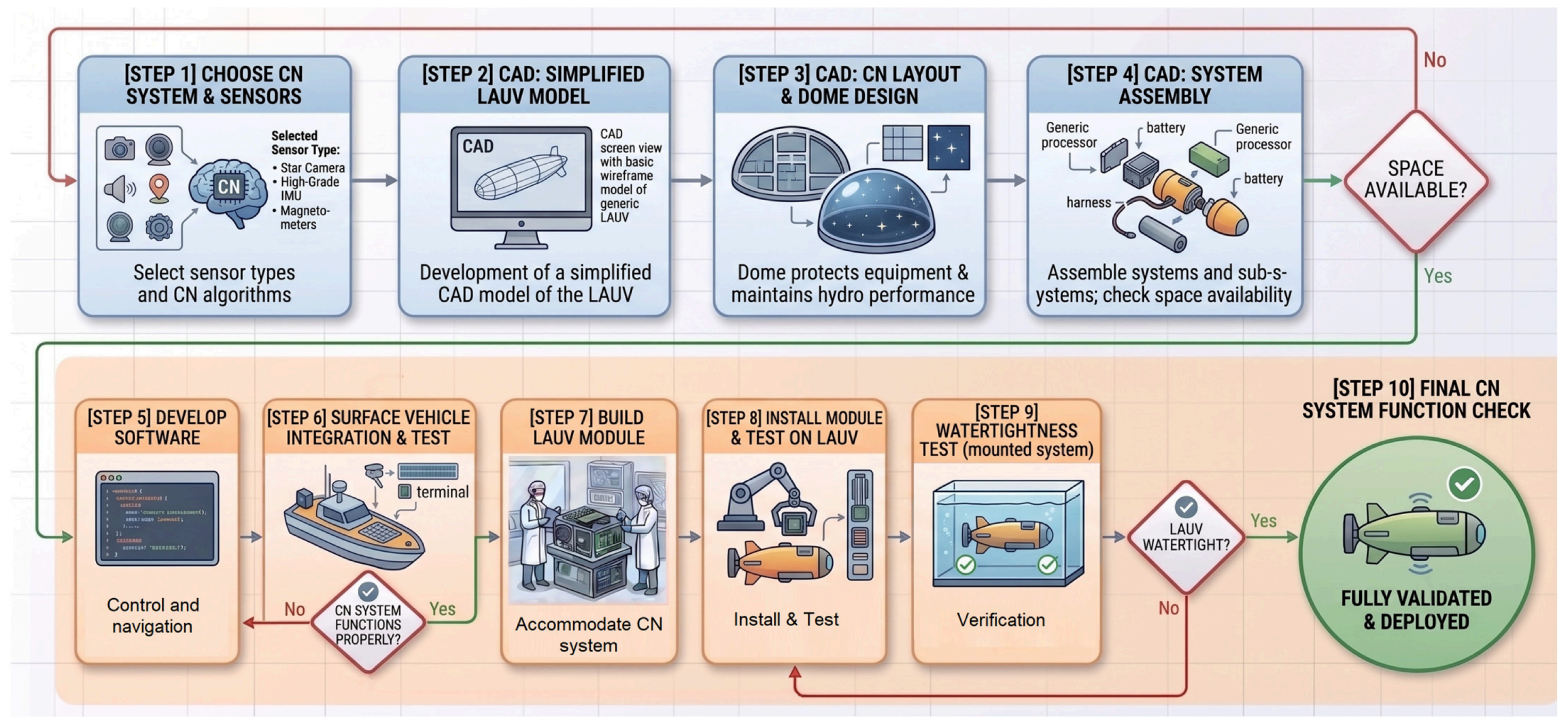
Methodology workflow for the conceptual development of CN system for the LAUV.

A methodology for the development of a CN conceptual application to the LAUV follows (see Figure 2).

The methodology may be described as follows:

- Step 1: Selection—Choose the appropriate CN system and sensors to apply. This phase requires evaluating the hardware against the constraints of the LAUV platform, prioritizing criteria such as volume, power consumption, pointing accuracy and daytime operational availability;
- Step 2: CAD Modeling I—Development of a simplified CAD model of the LAUV to establish the baseline spatial envelope. This ensures all internal bulkheads, payload bays and existing communication masts are accurately represented before introducing new hardware;
- Step 3: CAD Modeling II—Development of the CN layout design considering the LAUV’s space availability and the encapsulation (e.g., a dome) that should allow for celestial measurements while protecting the equipment and maintaining its hydrodynamic performance (to be verified in future work);
- Step 4: CAD Assembly and Verification—Assembly of the systems and sub-systems. A digital interference check is performed to ensure the selected sensor and dome do not clash with the vehicle’s structural frame. If there is no space available, go back to step 1 to select a smaller sensor or reevaluate the layout;
- Step 5: Software—Develop control and navigation software required to translate the raw celestial observations (e.g., solar azimuth and elevation) into an absolute georeferenced position that can be used by the LAUV’s navigation unit;
- Step 6: Integration and Testing—Install and test the CN system on a controlled environment, e.g., surface (water or land) vehicle and verify if the CN system functions properly under dynamic motion. If the CN system does not function properly, go back to step 5 to refine the algorithm or software integration;
- Step 7: LAUV Module—Build a physical LAUV payload module to accommodate the CN system, ensuring that the manufacturing tolerances align with the CAD simulations performed in Steps 3 and 4;
- Step 8: Installation and Testing—Install the module and test the integration (system compatibility, communications, power draw and data logging) of the CN system directly on the LAUV;
- Step 9: Watertightness—Test the watertightness of the system mounted on the LAUV using a pressure chamber or controlled pool environment to verify the integrity of the dome seals. If the LAUV is not watertight, go back to step 8 to address mechanical sealing failures;
- Step 10: Final Validation—Verify if the CN system functions properly in a more realistic and operational environment, specifically evaluating its ability to correct cumulative INS/DVL drift during surfacing maneuvers.

The main results of the conceptual system are presented in the following section.

## 4. Results of the CN Conceptual System

The sensor selection derived from a comparison of three hardware categories: low-cost vision-based implementations [5], miniaturized star trackers [22] and dedicated solar tracking sensors [23]. Vision-based approaches are the most accessible in cost terms but require custom software pipelines and achieve pointing accuracy of approximately 0.5 degrees. Star trackers offer the highest precision (0.002 to 0.01 degrees) but are confined to night-time or low-light conditions and demand more integration work. The Sunto’s STS was selected because it offers a useful balance, namely, a competitive unit cost of approximately 13,000 EUR, a plug-and-play Tracking Control Intelligence (TCI) interface that simplifies the software integration and a 0.01 degree pointing accuracy that aligns with the LAUV’s mission profile. It also represents a well-bounded starting point consistent with Step 1 of the ten-step methodology in Section 3. The accepted trade-off is daytime-only coverage in which pairing the STS with a star tracker to achieve 24 h positioning is a solution that should be addressed in future work.

It is important to clarify that, although Sunto rates the STS enclosure to withstand harsh environmental conditions, the sensor remains fundamentally optical. Hence, it needs an unobstructed view of the sun and cannot acquire a fix through dense cloud cover or heavy precipitation. The sensor’s main technical characteristics are detailed in Table 6.

The STS sensor should be connected to Sunto’s TCI [24] to receive sensor information and calculate the sun’s height and the Azimut between true North and the Sun’s position.

**Figure 3:**
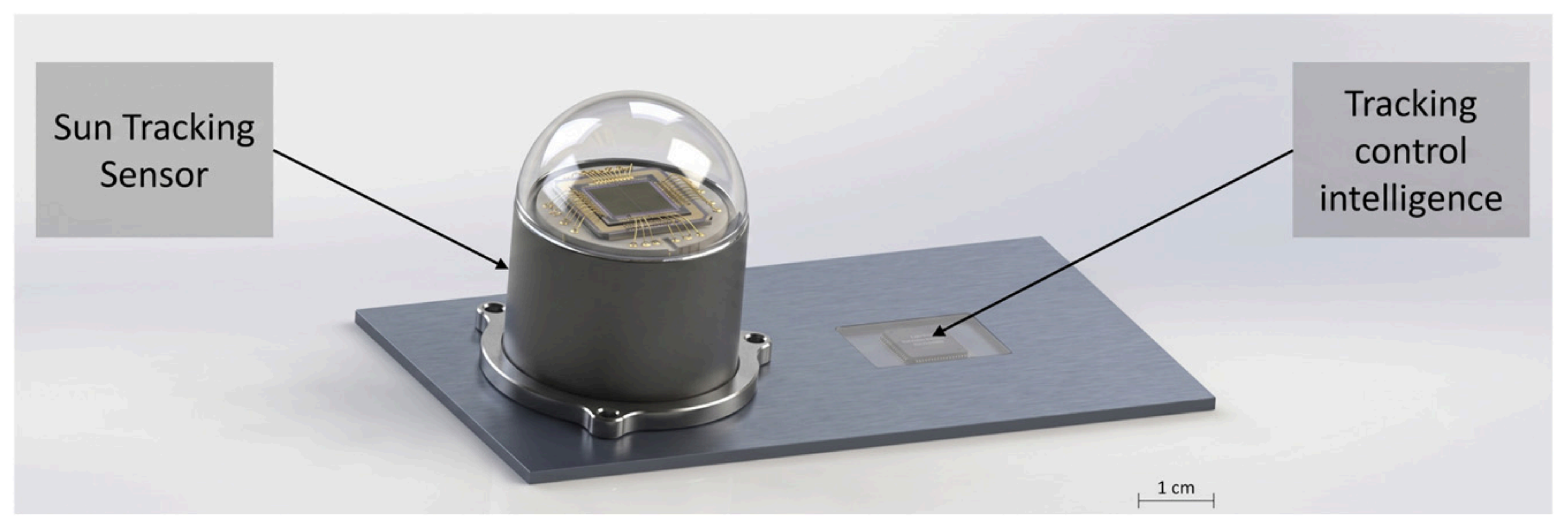
AI-generated rendering illustrating the CAD model of Sunto’s STS and TCI base and cover.

Following the methodology presented in Section 3, a CAD model of Sunto’s STS and TCI design base and cover is developed. Due to confidentiality restrictions, an AI-generated rendering is presented in Figure 3.

**Figure 4:**
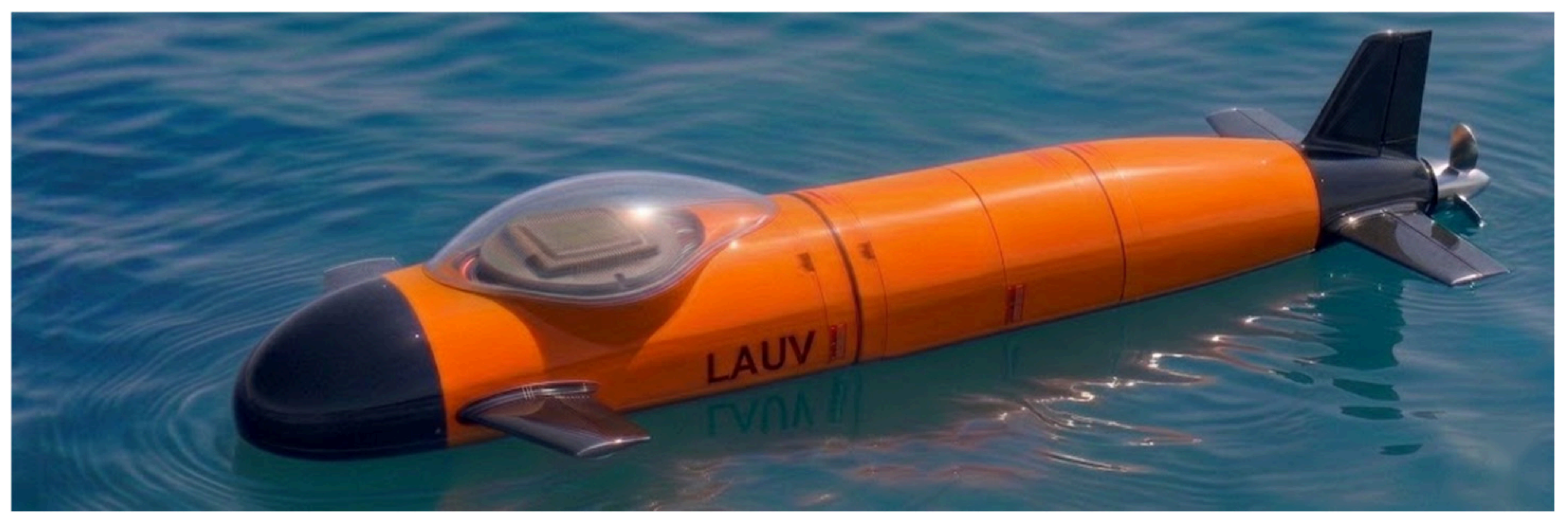
AI-generated conceptual rendering illustrating the proposed mounting arrangement for the observation dome and CN module on the upper hull of a LAUV.

This system enables GNSS-independent position estimation using celestial observations. It is then incorporated into the CAD model of the LAUV. Due to confidentiality restrictions regarding the actual engineering models, an AI-generated rendering is provided in Figure 4 to conceptually illustrate how this module might be physically arranged on the LAUV platform.

In practical terms, the CN module sits alongside the existing INS/DVL pipeline rather than replacing it. Each time the LAUV surfaces, the STS acquires a solar fix that resets the accumulated position error, after which, dead-reckoning resumes from a corrected baseline. The result is a navigation architecture that is genuinely independent of satellite infrastructure, with CN providing the absolute position anchors that INS/DVL alone cannot supply over long missions.

The results presented here represent the first steps in a structured conceptual development methodology. The CAD analysis illustrates that hardware integration is geometrically feasible. The remaining steps of Section 3 methodology, software development, bench testing, module fabrication, watertightness qualification and open-water validation, which are part of future work, provide a path from this conceptual baseline to an operational system.

## 5. Discussion

The selection of Sunto’s STS is supported by the literature review presented. The system’s precision, visibility dependence and autonomy requirements are consistently identified as critical for viable optical CN systems [4,5,18,19]. The system’s 0.01° pointing error, all-weather operational capability and auto-alignment functionality meet these requirements. The ten-year MTBF and competitive unit cost further justify the selection of the system within the practical constraints of an AUV research program. The integration of the STS [23] with the TCI unit [24] eases the computation of solar elevation and azimuth, making available the functional architecture needed for a georeferenced positional fix that is independent of satellite infrastructure. The result is a positioning system that needs no satellite contact [1,15,16] and that, ultimately, is what motivates this research.

Despite these functional advantages, implementing Sunto’s STS on an underwater moving platform presents inherent limitations. First and foremost, the sensor cannot operate while submerged as the LAUV must surface or employ a periscope, which breaks submerged mission continuity and limits deep-water applicability. Furthermore, the system’s reliance on optical visibility means that dense cloud cover, fog, or precipitation can block solar tracking entirely. Additionally, the required protective dome is highly vulnerable to the hostile marine environment. Sea spray, salt accumulation and biofouling can degrade optical clarity, while the sensor itself must survive continuous pressure cycling and hull vibrations. Finally, because the STS provides intermittent absolute fixes rather than continuous data, its readings are susceptible to noise induced by the physical instability of the AUV rolling and pitching in rough sea states. Addressing these limitations is required for transitioning this concept into a fully operational system and is part of future work.

Starting with solar tracking was a deliberate choice, not a limitation. This provides the project with a clearly defined starting point, in line with the step-by-step approach outlined in Section 3. The CAD integration, presented in Figure 3 and Figure 4, illustrates the geometric feasibility of accommodating the CN module within the LAUV’s compact hull without structural modification, a result that assesses the spatial assumptions made at the design stage. It should be noted that, while this spatial layout illustrates a possible physical implementation, it does not demonstrate hydrodynamic compatibility. The addition of a surface piercing dome will inherently alter the vehicle’s drag profile and flow characteristics. Formal verification through Computational Fluid Dynamics (CFD) and subsequent physical tank studies are required to quantify these effects and validate the integration.

It is worth putting the navigation benefit of a periodic CN fix into concrete numbers. A representative LAUV mission of six hours at 1.5 m/s covers approximately 32 km. At the INS/DVL drift rates of 0.1 to 1 percent of distance traveled reported in [2], uncorrected position error reaches 32 to 320 m by mission end, which is consistent with the multi-hour error accumulations described in [8]. A solar fix at surfacing, drawing on the STS’s 0.01-degree pointing accuracy and assuming a horizon reference error of around 0.1 degrees, resets that accumulated error and restarts the dead-reckoning clock from a corrected baseline. Even at a conservative fix rate of one surfacing per hour, this correction mechanism substantially bounds the worst-case navigation error across the full mission duration. That is the practical case for the hybrid INS/DVL/CN architecture proposed here.

The next step is to develop the software layer that translates raw STS/TCI output into a georeferenced position fix compatible with the LAUV’s navigation unit. Beyond that, testing within the GRU-PF [10] and RBF [14] filtering frameworks reviewed in Section 2.1 would aid quantifying the additional accuracy the can be extracted from each solar fix when the filter is given the opportunity to weight it appropriately against the continuous INS/DVL stream.

## 6. Conclusions and Future Work

This paper reviews automated CN technologies, mainly focusing on work published from 2020 to 2026, and assessed their suitability as a GNSS-independent positioning backup for AUV. The central finding of the SWOT analysis is that, despite real engineering challenges (e.g., optical access, biofouling, intermittent updates, etc.), CN occupies a position that no other currently available method does. It provides an absolute position fix from universally observable celestial bodies, requires no pre-deployed infrastructure and is not affected by GNSS jamming and spoofing. This combination makes it directly relevant to long-endurance AUV missions in contested environments.

Building on this foundation, a conceptual integration of a solar-based CN module into the LAUV platform was developed through the ten-step methodology introduced in Section 3. CAD modeling illustrates that Sunto’s STS and TCI can be accommodated within a protective dome on the vehicle’s upper hull without significant structural modification. This result consolidates the geometric assumptions behind the conceptual integration concept. The solar-centric approach was chosen for its clearly defined hardware, manageable integration pathway and continuous daytime fix capability. Extending that coverage to night-time and adverse weather conditions by adding a miniaturized star tracker is one of the next steps identified for future work.

It should be clearly stated that what is presented here is a conceptual design. The geometric feasibility of the integration has been illustrated, but the system has not yet been built, tested or validated in water. Moving from this stage to an operational capability will require software development, physical module fabrication, watertightness and pressure qualification, bench and tank/pool testing and ultimately open-water trials against a GNSS ground truth. These steps are well-defined and form the agenda for the work to follow. Namely:

- Hydrodynamic and Structural Verification: CFD analysis and mechanical stress testing are needed to formally verify that the proposed surface piercing dome does not introduce unacceptable drag or structural risk at LAUV operating speeds and depths;
- Hardware Development: An all-day integrated sensor combining the Sunto’s STS for daytime operation with a miniaturized star tracker for night-time and low-visibility conditions would extend the operational window. Also, anti-biofouling optical window design should be treated as a parallel hardware priority;
- Algorithm Integration and Simulation: The GRU-PF and adaptive EKF frameworks identified in Section 2.1 are natural candidates for integrating intermittent CN fixes with the continuous INS/DVL data stream. Dynamic simulation will be needed to quantify the achievable drift correction under representative mission profiles before committing to physical implementation.

Taken together, the trajectory is clear. Combining automated CN with INS, DVL and AI-based filtering offers a credible path to navigation that is resilient, independent of satellite infrastructure, resistant to electronic attack and applicable wherever the vehicle can surface long enough to take a fix.

## Figures and Tables

**Table 1:** Summary of standard AUV navigation technologies and auxiliary sensor inputs.

Technology/Sensor Category	Navigation Capabilities	Operational Challenges
Inertial and Dead-Reckoning (INS/DVL)	Provides continuous estimation of position, based on velocity, heading and attitude (roll, pitch, yaw).	Subject to cumulative time-varying drift; requires periodic external fixes to reset error.
Acoustic Positioning (LBL/USBL)	Determines absolute or relative position using fixed transponders or seafloor features.	Requires pre-deployed infrastructure; performance is limited by speed of sound variations and multi-path interference.
Geophysical and Optical (Sonar/Cameras)	Enables landmark-based localization, seafloor mapping and obstacle avoidance.	Highly affected by water turbidity, light attenuation and salinity/temperature gradients.
Auxiliary State Sensors (Pressure/Magnetic)	Provides absolute depth (*Z*-axis) and heading reference through pressure and magnetic field readings.	Vulnerable to magnetic interference and systemic errors during rapid surge accelerations.

**Table 2:** Highlights of recent works (2020–2026) on AUV navigation systems.

Authors and Year	Highlights
González-García et al., 2020 [9]	Review of localization, communication and navigation technologies; No universal best system exists; hybrid systems combining different types of sensors and AI are the key driver for future developments.
Lin et al., 2021 [10]	New method that combines Particle Filter (PF) with a Gated Recurrent Units (GRUs) deep neural network to improve state prediction; even in the case of short-term target loss, GRU Particle Filter (GRU-PF) can still achieve low error and stable estimations.
Tani et al., 2023 [11]	New navigation framework for AUVs that uses visual–inertial odometry that merges virtually derived velocity estimates with standard depth and altitude measurements; benchmark against DVL; experimentally validated.
Cheng et al., 2023 [12]	Novel navigation and control methods based on polarization patterns; hardware and software development; validated in real underwater environments.
Yu et al., 2023 [13]	Original navigation method that fuses seafloor topography and underwater magnetic fields; Extended Kalman Filter (EKF) is used to improve the integration of real-time topographic elevation and magnetic strength data with pre-existing marine geophysical maps; enables navigation with precision and autonomy; independent of the need to surface.
Ali et al., 2026 [14]	Novel approach that employs Radial Basis Function (RBF) deep neural networks to dynamically adjust the distance of its hidden neurons through weight adaptation; shown to enhance the accuracy of the estimated trajectory, position and velocity to a significant degree, proving highly effective under complex maneuvering conditions.

**Table 3:** Highlights of recent works (2020–2026) on CN.

Authors and Year	Highlights
Grm & Grm, 2021 [17]	Explores the potential of commercial smart devices as handheld CN systems, with a focus on addressing the limitations and errors inherent in human-centric navigational practices at sea.
Li et al., 2022 [19]	Development of an adaptively robust filtering algorithm, specifically designed for processing maritime CN data with the primary function of mitigating observation noise, thereby enhancing the overall accuracy of the system.
Venkat, 2022 [18]	Analyzes the challenges of overcoming human-centric observation errors and proposes achieving an autonomous celestial navigation system through the integration of automated observation devices and advanced algorithm-based processing similar to those used in spacecraft and aviation.
Critchley-Marrows & Mortari, 2023 [4]	Explores the modernization of traditional techniques by means of advanced models of celestial bodies and the horizon with the objective of automating and enhancing sextant-based maritime navigation.
Teague & Chahl, 2024 [5]	Development of a cost effective, vision-based algorithm for enhancing CN that enables continuous operation in environments where GNSS signals are jammed or unavailable.
Liubarets, 2024 [16]	Analyzes the need for CN as a fail-safe backup to GNSS, outlining how its integration enhances the overall reliability and safety of modern maritime navigation.
Lušić et al., 2024 [15]	Review on the contemporary relevance of CN with analysis of its transition from a traditional art to a critical, modernized component of future navigational resilience.
Sang et al., 2024 [22]	Reports on the successful application of a nano-star tracker for precise altitude measurement of the Chinese Advanced Space Technology Demonstration Satellite boom.
Wakita et al., 2025 [21]	Explores the conceptual design on the optimal FOV, required for automated CN camera systems intended for use on maritime navigation.
Sunto, 2026 [20]	Focuses on the development and application of dedicated solar tracking hardware sensors that are designed to continuously determine the position of the sun for automated systems.

**Table 4:** Comparative overview of autonomous CN hardware modalities.

Authors and Year	Volume (dm^3^)	Accuracy (◦)	Power (W)	Others
Polarization Sensor (Cheng et al., 2023 [12])	-	0.5	3	Day and night; operates submerged; require calibration for water column effects.
Miniaturized Star Tracker (Sang et al., 2024 [22])	0.5	0.0014	0.85	Daytime (clear horizon); degraded in rough seas; susceptible to water spray.
Solar Tracking Sensor (Sunto, 2026 [20])	1	0.01	5	Daytime; requires anti-biofouling window.

**Table 5:** SWOT analysis matrix of CN for AUV navigation in GNSS-compromised environments.

** S—STRENGTHS**	** W—WEAKNESSES**
**● GNSS-independent by design** CN derives position from celestial bodies, which means jamming, spoofing, or signal denial cannot affect it at any stage [5,15,16]. No satellite contact is needed. **● No pre-deployed infrastructure** Acoustic systems depend on transponder networks; CN does not. Stars and the sun are universally accessible, requiring no shore side setup and imposing no range constraint—a meaningful operational distinction noted in [8,9]. **● Well-established theoretical foundations** The astronomical and mathematical models underpinning CN have been refined over centuries and are thoroughly validated [4,15]. This stability benefits modern automation efforts: the navigation models themselves do not need to be re-derived, only implemented. **● Precision is improving with automation** Recent implementations combining star trackers, automated cameras and adaptively robust filtering have narrowed what was historically a significant accuracy gap [4,18,19]. **● A natural corrector for INS/DVL drift** Perhaps most relevant to AUV operations, CN can supply periodic absolute fixes to reset accumulated inertial navigation errors, fitting naturally into hybrid sensor fusion architectures [8,9].	**● Weather and visibility dependence** Cloud cover, fog and heavy precipitation can block solar or stellar observations entirely [15,16,17]. For a surface piercing system, this is the single most limiting factor in practice. **● Limited applicability in fully submerged operations** An AUV at operating depth has no line of sight to the sky. Observations require either surfacing or a mast/periscope arrangement, neither of which supports sustained covert or deep-water missions. **● Manual observation carries significant error rates** Historically, CN has been vulnerable to operator error. Reference [17] showed that even modern smart device implementations do not resolve this. Substantial operator-induced variability persists even in controlled conditions [18]. **● Relatively low update rate** Unlike INS or DVL, CN provides intermittent corrections rather than a continuous data stream, which limits its role to that of a periodic corrective input within a broader navigation architecture. **● Full automation demands sophisticated hardware** Star trackers, optimized camera FOV designs and machine vision pipelines are required [4,21]. Getting this hardware small and robust enough for AUV integration remains an open engineering problem. **● Horizon-referenced measurements accumulate errors** Altitude fixes referenced to the sea horizon are affected by atmospheric refraction, by the horizon’s physical instability in rough sea states and the natural light level required by the sensors. Advanced compensation modeling is needed [4].
** O—OPPORTUNITIES**	** T—THREATS**
**● Full automation is within reach** Camera sensors and star trackers comparable to those used in aerospace are being actively adapted for maritime CN [4,5,18] and the performance gap between manual and automated observation is closing rapidly. **● CN fixes can slot into existing AI/Machine Learning frameworks** The GRU-PF [10] and RBF deep neural network [14] frameworks reviewed here already handle heterogeneous sensor inputs. A CN-derived absolute fix could be incorporated without requiring fundamental architectural changes. **● Solar tracking extends the operational window** Dedicated solar tracking hardware [20] provides continuous daytime positioning, supplementing star-based techniques that are limited to night-time or by overcast conditions. Together, these cover a wider operational window than either approach alone. **● GNSS denial is increasingly a design requirement** Military and critical infrastructure missions are treating GNSS availability as unreliable by default [5,15,16]. CN is one of few positioning methods entirely independent of satellite signals, raising its strategic relevance. **● FOV research transfers directly to AUV configurations** The conceptual work by Wakita et al. [21] on optimal camera FOV design for maritime autonomous surface ships used geometries and assumptions directly applicable to periscope or mast-mounted AUV installations. **● Budget-accessible implementations exist** CN algorithms developed for cost-constrained platforms [5] demonstrate that viable CN does not necessarily require custom-made hardware, which is an important consideration for research programs with limited procurement budgets.	**● Biofouling and adverse weather can disable the system for extended periods** Persistent cloud cover, sea spray and biological fouling of optical surfaces are realistic operational failure modes, not edge cases. Any deployment must engineer against prolonged sensor unavailability. **● Competing GNSS-independent methods do not require surface access** Geomagnetic navigation [13] and polarization-based techniques [12] operate fully submerged and may prove better suited to AUV platforms. CN will need to demonstrate clear advantages over these alternatives, not just over GNSSs. **● Infrequent fixes can weaken sensor fusion filters** Under dynamic maneuvering, a delayed or missed CN fix may introduce latency or divergence in fusion filter state estimates, which is a system-level integration risk that must be assessed. **● Marine environments are hostile to optical hardware** Salt water, pressure cycling, vibration and biofouling impose stringent qualification requirements on star trackers and optical sensors. Achieving the robustness needed for operational AUV deployment is a non-trivial engineering challenge. **● No certification framework is yet in place** Standardization for autonomous CN systems in maritime applications is still in early development. Deploying CN as a primary navigation mode currently carries regulatory uncertainty that could complicate operational approval. **● Physical obscuration is a threat GNSS-independence cannot prevent** In adversarial scenarios, optical sensors remain vulnerable to smoke, laser blinding, or directed obscuration. This is a threat class that does not affect INS or acoustic systems and that deserves consideration in contested environment mission planning.

**Table 6:** Sunto’s Solar Tracking Sensor advantages and details [23].

#	Advantage	Detail
1	Low sun direction error	Pointing error of 0.01°
2	Wide viewing angle with high pointing accuracy	Facilitates rapid solar acquisition without requiring precise initial platform alignment
3	Auto-alignment of the solar tracking system	Ensures high precision in continuous sun tracking
4	Easy installation and extremely low maintenance	—
5	High reliability	Mean Time Between Failures (MTBF) ≈ 10 years
6	Competitive pricing	Approximately 13,000€

## Data Availability

Data is contained within the article. CAD models are unavailable due to confidential restrictions.

## Abbreviations

The following abbreviations are used in this manuscript:

AI	Artificial Intelligence
AUV	Autonomous Underwater Vehicle
CAD	Computer-Aided Design
CFD	Computational Fluid Dynamics
CN	Celestial Navigation/Astronomic Navigation
DVLs	Doppler Velocity Logs
EKF	Extended Kalman Filter
FOV	Field of View
GNSSs	Global Navigation Satellite Systems
GRU	Gated Recurrent Unit
GRU-PF	Gated Recurrent Unit Particle Filter
IMU	Inertial Measurement Unit
INSs	Inertial Navigation Systems
LAUV	Light Autonomous Underwater Vehicle
LBL	Long BaseLine
LSTS	Underwater Systems and Technology Laboratory
MTBF	Mean Time Between Failures
PF	Particle Filter
RBFs	Radial Basis Functions
STS	Solar Tracking Sensor
SWOTs	Strengths, Weaknesses, Opportunities and Threats
TCI	Tracking Control Intelligence
USBL	Ultra-Short Baseline
UUV	Unmanned Underwater Vehicle
